# Report on the Progress of Dentistry

**Published:** 1882-01-20

**Authors:** T. A. Chandler


### REPORT ON PROGRESS OF DENTISTRY.

BY T. A. CHANDLER, D. M. D.

Dentistry, in its progress, seems to have come to a pause in the direction in which for many years it has been so active, namely, in operations on the teeth and the manipulation of gold, and to have turned back to take up again the long neglected and despised mechanical methods and appliances. Some few are still at work on the minute microscopy of the tooth substance, and claim to have discovered, under the guidance of Heitzmann, a living network branching in every direction and penetrating the formed material, uniting the offshoots from the nerve substance or tooth pulps, the soft solids as they are often called, and explained in this way the mysterious sensitiveness of dentine, which has up to the present so puzzled all workers on the teeth, and claim to show how the tooth itself is built up and nourished.

Legros and Magitot in France have investigated the tooth follicle, and their original memoir has been done into English with additions, fitting it for an elementary text-book, by Dr. M. S. Dean of Chicogo. Kingsley's Oral Deformities has been added to the literature on this side of the Atlantic, and on the other several books of less pretension, chiefly upon mechanical dentistry. Several new dental magazines have come into existence, without adding much to the quality, but a great deal to the bulk, of the reading matter which every progressive man is expected to peruse. The Dental Cosmos still keeps the lead of the magazines, and within a short period has much improved in the quality of its articles.

As was said at the outset, operative dentistry has apparently come to a pause in its career, and the army of practitioners is dividing its force: "Some for Paul and some for Apollos;" some are looking back to the old-fashioned soft foil, and the ancient methods, as though all that is new is vanity and "there is nothing new under the sun;" still others seem to think all that is old is worthless, and nothing of value that is not "brand new." Such adhere to the use of cohesive gold, or follow the leaders of the "new departure." whose gods are amalgam and other plastic materials; who declare that "in proportion as teeth need *saving*, gold is the *worst* material to use;" and that "a filling may be the *best* known for the tooth and yet *leak badly.*" (Italics not mine.) As long as these varying methods are in the hands of thoughtful men, some good may come out of the evil, and the pause may be but the precursor of another era of progress. Skepticism leads to inquiry as well as the bold assertion of startling propositions. Men will at last be brought to examine and compare, and he that is honestly seeking the best way for himself and his patients, who is not a partisan, will soon eliminate what of his own is faulty, and adopt what he finds good in his neighbor's.

Partnership is the bane of all true progress, and the differences of opinion seem so far to have tended in this direction. Especially is this true with regard to the advocates and opponents of the "new departure." Arguments give way to recrimination, assertions are made to stand for proofs. So startling is the assertion made by the adherents of this new school, namely, that "since the very day of the birth of dentistry its practitioners have been on a wrong scent, have been, and still are, ruining teeth instead of saving them," it is little wonder that amazement and indignation should have been the first feeling awakened, that wild assertion should have been met by as wild contradiction, and as wild counter-assertion. Just now the battle seems to be hushed, and the combattants are looking for results. The truth seems to be that, like all hobby-riders, the riders of this new horse drove him too hard at the beginning. What there was of good in their claims is not new, and what is new is not all good. At the bottom of their claims is the galvano-electric theory, which has yet to be proved; and until this is done it would seem of little use to build theories or methods upon it.

Mechanical dentistry, which has so long held a subordinate place, seems now coming to the front again. New ways of working old materials, and new operations, are being constantly brought forward and claiming the attention of the best men. Of the new materials, celluloid is holding its own against vulcanite, and the controversy over the latter seems to have resulted in giving us a really valuable base. The "new mode" of working it promises to revolutionize its character, and to render it, instead of an unreliable and poor material, one of the most useful in our possession, almost a new thing. Even vulcanite, which all thought had reached its climax, feels the impulse of this "new mode," and with it better results are reached than ever before. Continuous gum work, as it is called, consisting of a platinum plate, entirely covered on its buccal and lingual surfaces with a veneer of porcelian, imitating in its color that of the natural membrane, feels the boom, and better ways of making it and improved material have made it the fashionable base for whole sets of artificial teeth. The setting of "pivot teeth," formerly confined to the six front upper teeth, is now by new methods extended to the bicuspids, and even to the molars, enabling good roots to be saved and made useful that were formerly worse than useless, freeing the patient from the necessify of a plate. It is safe to say, that by one or the other of these methods, more roots are now saved and made useful than at any previous period in the history of dentistry.—[Boston Medical and Surgical Journal.

---

**Method of Applying Nitric Acid as a Caustic.**—To prevent the spreading of nitric acid when used as a caustic, Dr. Speirs recommends, in the Practitioner, that the part to be cauterized, as for example, a nævus, should first be surrounded by a short glass tube or neck of a bottle, of the size of the slough desired, and then the nitric acid applied with a pipette. Before removing the tube, the excess of acid is to be mopped up with cotton-wool on a probe.—[Med. News.

